# KIT 1 (Keep in Touch) Project—Televisits for Cancer Patients during Italian Lockdown for COVID-19 Pandemic: The Real-World Experience of Establishing a Telemedicine System

**DOI:** 10.3390/healthcare11131950

**Published:** 2023-07-06

**Authors:** Calogero Casà, Barbara Corvari, Francesco Cellini, Patrizia Cornacchione, Andrea D’Aviero, Sara Reina, Silvia Di Franco, Alessandra Salvati, Giuseppe Ferdinando Colloca, Alfredo Cesario, Stefano Patarnello, Mario Balducci, Alessio Giuseppe Morganti, Vincenzo Valentini, Maria Antonietta Gambacorta, Luca Tagliaferri

**Affiliations:** 1 Fatebenefratelli Isola Tiberina-Gemelli Isola, Via di Ponte Quattro Capi 39, 00186 Rome, Italy; calogero.casa@fbf-isola.it; 2 Fondazione Policlinico Universitario A. Gemelli IRCCS, Largo A. Gemelli 8, 00168 Rome, Italy; barbara.corvari@policlinicogemelli.it (B.C.); francesco.cellini@policlinicogemelli.it (F.C.); giuseppeferdinando.colloca@policlinicogemelli.it (G.F.C.); alfredo.cesario@policlinicogemelli.it (A.C.); stefano.patarnello@guest.policlinogemelli.it (S.P.); mario.balducci@policlinicogemelli.it (M.B.); vincenzo.valentini@policlinicogemelli.it (V.V.); mariaantonietta.gambacorta@policlinicogemelli.it (M.A.G.); luca.tagliaferri@policlinicogemelli.it (L.T.); 3 Mater Olbia Hospital, SS 125 Orientale Sarda, 07026 Olbia, Italy; andrea.daviero@materolbia.com; 4 Dipartimento di Scienze Radiologiche ed Ematologiche, Università Cattolica del Sacro Cuore, Largo Francesco Vito 1, 00168 Rome, Italy; sara.reina@guest.policlinicogemelli.it (S.R.); silvia.difranco@guest.policlinicogemelli.it (S.D.F.); alessandra.salvati@guest.policlinicogemelli.it (A.S.); 5 Department of Experimental, Diagnostic and Specialty Medicine, Alma Mater Studiorum University of Bologna, Via Zamboni 33, 40126 Bologna, Italy; alessio.morganti2@unibo.it; 6 IRCCS Azienda Ospedaliero-Universitaria di Bologna, Via Giuseppe Massarenti 9, 40138 Bologna, Italy

**Keywords:** telemedicine, digital health, radiation oncology

## Abstract

To evaluate the adoption of an integrated eHealth platform for televisit/monitoring/consultation during the COVID-19 pandemic. Methods: During the lockdown imposed by the Italian government during the COVID19 pandemic spread, a dedicated multi-professional working group was set up in the Radiation Oncology Department with the primary aim of reducing patients’ exposure to COVID-19 by adopting de-centralized/remote consultation methodologies. Each patient’s clinical history was screened before the visit to assess if a traditional clinical visit would be recommended or if a remote evaluation was to be preferred. Real world data (RWD) in the form of patient-reported outcomes (PROMs) and patient reported experiences (PREMs) were collected from patients who underwent televisit/teleconsultation through the eHealth platform. Results: During the lockdown period (from 8 March to 4 May 2020) a total of 1956 visits were managed. A total of 983 (50.26%) of these visits were performed via email (to apply for and to upload of documents) and phone call management; 31 visits (1.58%) were performed using the eHealth system. Substantially, all patients found the eHealth platform useful and user-friendly, consistently indicating that this type of service would also be useful after the pandemic. Conclusions: The rapid implementation of an eHealth system was feasible and well-accepted by the patients during the pandemic. However, we believe that further evidence is to be generated to further support large-scale adoption.

## 1. Introduction

The COVID-19 pandemic had, and it is having, a significant and heavy impact on everyday life, economies on a global scale and, of course, the global health system [1,2]. At the beginning of the pandemic, the statistics were so serious that the hospital system had to reorganize radically and promptly in order to improve the safety of patients and staff while continuing to provide essential services [3,4,5,6,7,8,9,10].

Recently and in a short amount of time, national and international oncological scientific societies provided guidelines or suggestions to deal with the need to both treat cancer patients affected by malignancies and to limit unnecessary contact to reduce the risk of contagion [11,12,13,14,15,16].

In that unexplored clinical scenario, digital health technology played a role on several levels [17], from big data and artificial intelligence (AI) applications [18,19,20] to the development of dedicated tool for contact tracing and for COVID-19 testing and tracking [18,21,22].

In particular, eHealth protocols were suggested to facilitate communication between patients and health professionals; access to health resources; and the organization, interpretation, and dissemination of health data [23].

The last subset of the eHealth revolution is mobile health care (mHealth), which permits mobile-based platforms and mobile applications to deliver health information through the internet [24]. Such applications can be accompanied by internet of things (IoT) devices such as wearable devices that, possessing vital parameter detectors, can help provide quantitative information about the patient that until now had not been fully considered in our clinical activities.

One of the widely explored areas of digital health and mainly implemented in clinical practice during the pandemic spread was telemedicine [25,26,27,28,29], especially for patients more vulnerable to exposure to infections (e.g., patients with a previous diagnosis of cancer and patients who have undergone immunosuppressive treatments such as chemotherapy or radiotherapy). For this reason, a particular interest quickly grew in the possibility of replacing outpatient follow-up with visits based on telemedicine.

The proposed use of digital technologies to support cancer patients’ care pathway is not, per se, an issue born out of the pandemic. The World Health Organization (WHO), for the 2019–2023 triennium, has encouraged the use of digital technologies to enable people to access the information and services they need to improve their resources throughout their lives [30]. However, as the study experiences of Basch et al. document [31,32,33], the use of electronic tools to monitor cancer patients during treatment has also been shown in randomized clinical trials to be a determining factor in a reduction of emergency room admissions, regardless of the patient’s computer experience [31]. A subsequent analysis of the same study also reported an advantage in terms of overall survival [32]. In another randomized clinical trial, the same research group demonstrated an advantage in terms of function, symptom control, and quality of life in the group randomized to the use of digital monitoring [33]. In this context of scientific knowledge related to digital monitoring of cancer patients, the COVID-19 pandemic has been a major event not only leading to higher mortality and morbidity in cancer patients than in the general population, but also seeing a reduction in the screening and diagnosis of malignancies and a delay in treatment and access to care pathways [34]. Although the overall effects of this delay have not yet been specifically measured and reported, it is estimated that this may contribute to increased mortality from malignancies in the near future [34].

However, it is also acknowledged that COVID-19 has led to a more rapid and widespread introduction of telemedicine tools in the clinical environment that would otherwise not have been so quickly introduced into everyday cancer care pathways [35,36].

In this context, the use of advanced telehealth systems capable of collecting PROMs (patient-reported outcome measurements) and PREMs (patient-reported experience measurements) represents an innovative and valuable opportunity to monitor the patients in their daily life and capture the evolution of their disease early, in addition to establishing a relationship of proximity and the positive perception of taking care [37]. PREMs are defined as a gathering of patients’ views of their experience whilst receiving care and represent an indicator of the quality of patient care.

To make explicit the context in which teleconsultation technology is placed in our center, it is useful to mention that the pandemic allowed professionals to introduce tools that could be applied within specific clinical research protocols and were capable of offering a new model for the monitoring and early diagnosis of COVID-19 infection. In our experience at the Radiotherapy Unit of the Fondazione Policlinico Universitario Agostino Gemelli IRCCS, known as “Gemelli ART” (Gemelli Advanced Radiation Therapy), in parallel with the experience of remote visits, an application for mobile devices was developed to collect biometric data which was recorded via innovative tools and then analyzed and integrated with artificial intelligence systems. Those tools were applied during the treatment of patients and the follow-up period via the use of a dedicated mobile application and, in another branch of same protocol, for the remote monitoring of the symptoms of healthcare professionals. Patients and healthcare professionals had the opportunity to communicate their status by answering questionnaires that were offered to them through the mobile application, which could, if necessary, be connected to a wearable device that collected daily real world data (e.g., daily steps, hours of sleep, and heartbeat). The huge amount of real world data collected, which was integrated with health data, would be translated into useful information for medical practices. Specific questionnaires could be provided to the patients through the mobile application, using new questions developed by a clinical team or standardized questions from scientific literature. The technology, designed in response to the state of emergency caused by the pandemic, could also be used in a non-emergency period to manage the daily life of radiotherapy patients and their toxicity levels. Moreover, via this monitoring system, a range of information was collected, such as distance traveled each day, steps walked, and blood oxygen saturation, to evaluate whether those items should be considered valuable in the early detection of possible COVID-19 infection or to predict toxicity or compliance outcomes for radiotherapy patients. All the collected data were stored interactively within the hospital electronic medical records, allowing further analysis to determine eventual association between the collected parameters and radiation treatment-related toxicity during the course of treatment in radiation therapy and in the early follow-up period.

The same health protection modalities during the COVID-19 pandemic were proposed to health care personnel who, caring for a high volume of patients on a daily basis while adopting all kinds of procedures designed to prevent infection, were potentially exposed to infection. This modality enabled the Radiotherapy Service to safely ensure continuity of treatment for ongoing patients.

The value of data accumulated in this way would be unremarkable without technology capable of analyzing it. Therefore, a dedicated facility equipped with adequate computing power and dedicated staff in computer science and data management was established in our center [37]. Within this facility, it is also possible to analyze the data obtained via artificial intelligence algorithms in order to study correlations, to develop predictive models, to identify recurring patterns, and to devise innovative solutions to enable physicians to quickly and effectively access data accumulated via mobile applications or to directly and automatically include such information in hospital databases.

The description of those tools, although they represent a closely related issue both in terms of the needs it aspires to meet and of the technologies adopted as a response to the needs of patients and health care providers, is not the focus of this article.

The aim of this paper is to report on monocentric real-world experiences with the application of a telemedicine system organized by managing a high volume of clinical visits using televisits or phone calls and email on the basis of patient risk category during the Italian lockdown period in a single radiation oncology center.

## 2. Materials and Methods

A multi-professional working group was tasked with modifying the usual care service in order to reduce patients’ exposure to COVID-19 and take advantage of the best resources available (workforce, facilities, equipment).

Specifically, new procedures were considered for the management of cancer patient visits to the Radiation Oncology Department of the Fondazione Policlinico Universitario Agostino Gemelli IRCCS in order to improve safety as much as possible.

The working group was composed of radiation oncologists, nurses, radiation oncology residents, and radiotherapy technicians (RTT).

In our institution, patients usually meet these professionals during treatments: every day, a nurse greets each patient who is to undergo radiation treatment on a daily basis by recording any clinical updates and reporting them, if necessary, to the referring physician; the RTT staff positions the patient appropriately for proper treatment, performs necessary and preparatory checks for treatment, and—except in cases requiring express medical authorization or unless there is an additional need—treats the patient; periodically, the radiation oncologist, depending on the type of treatment, on the dose achieved, on the established protocol, and on the possible occurrence of toxicity, visits the patient during scheduled therapy. The multi-professional group was established to ensure that each decision was made based on the specific needs of each patient and on specific clinical context. Specifically, it was necessary to focus on efficacy, feasibility, and, at the same time, considering the patient’s age, clinical condition, type of disease in terms of primary sites and stage, and personal needs and values.

Moreover, each choice needed to be clearly documented, especially in cases of deferred visits.

Since these actions were organized within clinical practice in response to pandemic needs and not for research purposes, it was not necessary to evaluate this study within a research protocol for submission to the ethics committee.

The visits scheduled in accordance with this scheme covered the period of the Italian lockdown, from 8 March to 4 May 2020. The new defined procedure allowed all planned visits to be distributed based on an evaluation of each patient, considering type of primary disease, stage, time since diagnosis, treatments performed, and recent follow-up radiological and/or laboratory examinations performed by the patient, according to the following scheme:

1 = visit not deferrable and kept in person (e.g., patients who needed to undergo radiation treatment)

2 = visit postponed

3 = visit performed via email and phone call management (e.g., low-risk follow-up)

4 = visit performed via complete teleconsultation using teleconsultation system

All visits preparatory to the initiation of radiation treatment, thus necessitating a clinical evaluation with physical objectivity possibly accompanied by operations that would have to be performed inside the hospital anyway (e.g., acquisition of a radiation therapy simulation using CT or MRI scans), were considered nondeferrable and therefore held in person. Similarly, all follow-up visits where, because of elements related to the specific pathology (e.g., risk factors), the treatments performed (e.g., particularly high risk of toxicity), or the appropriateness of a key contribution of the in-person physical objective examination was such that it could not be delegated to any family physician colleagues closer to the patient—especially in the case of patients who come from cities and regions other than our center—were held in person.

Visits in which the patient demonstrated negativity at follow-up after a period of time at a considerable distance from the last active treatments performed or in cases of sufficiently low-risk disease based on evaluation by the multidisciplinary task force were recommended for postponement. In such cases, the patient was offered the opportunity to send any laboratory or instrumental tests via email and to carry out a telephone interview with the referring physician.

In the specific situations where such telephone and/or e-mail contact was considered insufficient but, because of the pandemic and given the infection risk, the criteria for unequivocal need for an in-person conducted clinical examination were not met, teleconsultation was offered to the patient.

All patients were given a free choice whether to accept the remote visit or insist on an in-person clinical assessment.

The working group also searched the instrumentation necessary to organize teleconsultation visits. To purchase the teleconsultation material, the support of a patients’ association, “Associazione Attilio Romanini”, was requested. The Association accepted the project and provided for the purchase of the equipment within 10 days, allowing the visits to be rapidly rescheduled with teleconsultation. The project was called KIT (Keep in Touch). The software used for teleconsultation was Microsoft^®^ Teams. All patients who underwent a remote assessment managed via email and telephone call or teleconsultation gave verbal consent by telephone to the visit mode, while all patients who refused this method were visited in person.

At the end of the first month of teleconsultation system usage, all patients managed via the new system were contacted and subjected to a structured telephone interview composed of three questions to evaluate the level of satisfaction with the service and to investigate fears and perceptions. All described phases of the previously described project are summarized in Table 1. The items included in the questionnaire administered to the patients are listed in Table 2. The responses were analyzed quantitatively using the tools of classical descriptive statistics with 5-point Likert scale parameters. The primary outcome of the study is to report the real-world experience related to the establishment of a teleconsultation service in a single center during the COVID-19 pandemic; the secondary outcome is to collect patient-reported outcomes structured as a 5-point Likert scale telephone questionnaire.

## 3. Results

During the period of Italian lockdown (from 8 March to 4 May 2020) a total of 2027 visits (with 2013 different patients) were planned in the Radiation Oncology Department of the Fondazione Policlinico Universitario Agostino Gemelli IRCCS. Of this amount, 71 visits (3.50%) were cancelled due to patient death or patient decision to pursue radiotherapy nearby in his/her hometown.

**Figure 1:**
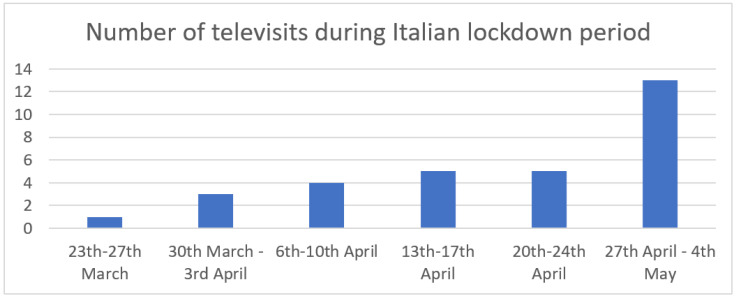
Number of televisits during Italian lockdown period.

**Figure 2:**
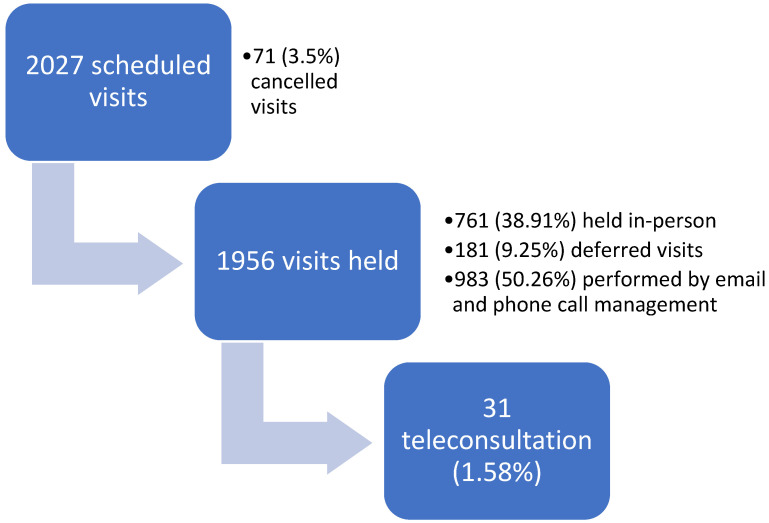
Flow diagram of visits scheduled during the Italian lockdown period (from 8 March to 4 May 2020).

From the evaluation of the remaining 1956 visits according to the previous described criteria, 761 (38.91%) were considered not deferrable and were held in person; 181 (9.25%) were postponed; 983 (50.26%) were performed via email management and phone call, while 31 visits (1.58%, with 30 different patients) were conducted using the video-teleconsultation system, as shown in Figure 1 and Figure 2.

**Figure 3:**
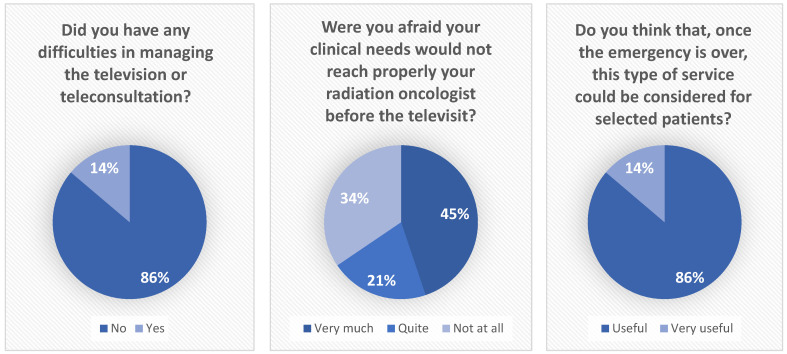
Patient-reported outcomes measures (PROMs).

A total amount of 664 min of video-teleconsultation was performed, with a mean value of 23 min for each one. Among the 30 patients who used the video-teleconsultation service, 29 responded to the telephone interview. The age of the patients was between 34 and 87 years old (median age 63) and 12 patients were >65 years old (41.3%); 17 patients were female (55.2%) while 13 patients were male (44.8%). During the interview, 4 patients (13.8%) declared difficulties in managing the video-teleconsultation and needed to schedule a new date for the televisit or managed the televisit using email and phone call; the other 25 patients (86.2%) declared themselves satisfied or very satisfied with the video-teleconsultation system. Among the 4 patients who reported difficulty in connecting, 2 were over 65 years of age. Thirteen patients (44.8%) declared themselves afraid or very afraid due to the fact that the pandemic may hinder their on-site clinical visit; 10 patients (34.5%) were not or not totally afraid; 6 patients (24.1%) were intermediately afraid. All 29 interviewed patients declared that, for selected patients, a video-teleconsultation service would also be helpful after the pandemic for the management of follow-ups related to their oncologic condition and disease if such a service was considered adequate by the referring healthcare team. Patient-reported outcomes measures (PROMs) are shown in Figure 3.

## 4. Discussion

A growing interest in telemedicine has already been reported even in oncology, with encouraging results in both retrospective studies and clinical trials [38,39]. Specific experiences were described especially in radiation oncology regarding treatment planning and for PROMs collection [40,41].

The spread of COVID-19 has catalyzed the sharing of experiences previously established in the field of telemedicine [42] and promoted the proposal of new solutions [43,44,45]. Multiple experiences documented in the literature, in particular, link the emergence and spread of telemedicine technologies and tools to the new and unforeseeable needs created by the pandemic [35,36]. Overall, digital health can offer cancer patients innovative services and assistance tools that were previously unimaginable [46,47,48,49,50]; indeed, the application of modern digital technology in oncology could lead to:

- Patient stratification and -omics predictive models [51,52,53,54,55,56,57,58,59,60,61,62,63,64];
- Toxicity and objective cosmetic outcomes evaluation after treatments [65,66,67];
- Data mining [68,69,70,71,72] and process mining [73,74,75,76,77];
- Digital tools to ensure proximity to the patient [50,78,79,80].

These new tools, progressively introduced into the technological field, were quickly dedicated to managing the challenges of the first pandemic of the contemporary era [17,81]. In parallel, digital devices and software capable of incorporating artificial intelligence algorithms are finding increasing scope in speeding up highly repetitive and time-consuming tasks for physicians. Such software can, for example, assist in the contouring of organs at risk, optimize an already developed treatment plan, or alternatively obtain an automated plan [82].

Moreover, over the past few months, digital technology to include other new artificial intelligence tools, has been embedding itself in every single aspect of the cancer patient care pathway and is also beginning to be used not only as an accessory capable of performing dedicated functions, but also as a potential substitute for processes currently the prerogative of human intelligence: for example, ChatGPT (Open AI, San Francisco, CA, USA) is considered fairly accurate in providing information related to oncology issues [82] and may even write a discharge letter for the patient [83]. Such technology carries with it risks related to personal data management [84]; the need for a quality control system capable of supervising, identifying any errors, and correcting them (safety by design); and the need to educate the personnel who will be working with such technology (safety by education) [85]. In the face of these scenarios of extreme digitization, the centrality of the doctor–patient relationship must be recognized to effectively make technological innovation ancillary to the patient care pathway, acknowledging an additional role not vicarious by technology of providing the human return that the patient demands from the physician and health care personnel with whom he or she interacts [86]. Such human needs also emerge in the contexts of high-tech treatments to the extent that in some cases they determine the need for true multi-professional and multidisciplinary task forces designed to ensure a “humanity assurance protocol” [87,88,89]. In this context, digital technology—used appropriately by patients, doctors, nurses, RTT, and other health care personnel—can also offer synergistic help to the processes of humanization of care [79,80].

In our experience, teleconsultation was also positively received by patients aged >65 years, who accounted for 40% of the scheduled televisits, and, although the sample of the present study is small, it does not appear that age was the factor most associated with difficulties in connecting and participating in the service; this opens up the discussion on the fact that, even in elderly patients, technology can be used, especially in cases of autonomous patients who are already accustomed to its use or caregivers who are particularly present. The main limitation of the study is the low number of patients involved in video-teleconsultation, for a total of 1.58% among all the visits scheduled; other limitations are the mono-institutional setting and the limited period of study. An additional selection bias may be the fact that teleconsultation has been offered in our center to more patients than have actually used it by always ensuring, should the patient request an in-person visit, that they can opt for in-person clinical evaluation. This could lead selected patients to be more predisposed to positively accept the hypothesis of managing the visit through a teleconsultation by obtaining higher appreciative responses than the total number of patients to whom it would have been initially proposed.

Although it seems that telemedicine in oncology will endure longer than the diffusion of COVID-19, in order to borrow for clinical practice the technological and organizational achievements developed due to pandemic needs [90], further attention has to be dedicated to the warnings provided by others’ academic experiences. Using an expressive quote reported by Tobias Finazzi et al. [91], in-person radiation oncology visits are “more than a pat on the back”. In our experience since the end of the lockdown, the number of teleconsultations has not fallen drastically. On the contrary, we assisted in a gradual settling, suggesting that remote assessment modality can become complementary to in-person clinical settings in the care pathway for selected cases and in particular contexts. In the field of teleconsultation, Italian national recommendations were rapidly provided to allow minimal technical requirements and general standards and tools to be defined [92]. However, in order to integrate opportunities for remote digital assessment systematically and safely into the clinical pathways of any pathology, it would also be interesting to collect evidence showing that it is not inferior to a face-to-face visit in certain well-defined clinical scenarios. In this regard, the low number of patients that underwent televisits may reflect, rather than a lack of confidence on the part of patients—who reported a considerable level of satisfaction (86.2%) when involved in teleconsultation evaluations—the absence of consideration of this service in cancer disease management guidelines. This opens up three aspects that need to be explored in the future: (i) the indications to propose a remote teleconsultation instead to an in-person visit to the patient; (ii) a new semeiotic to define areas of appropriateness for each telemedicine feature or component (digital application, webmail communication, video-teleconsultation etc.); (iii) the needs to establish a dedicated training in digital health and telemedicine [85].

The authors consider the results of this experience to be positive. In fact, as shown in Figure 1, after sporadic use of teleconsultation, there was a dramatic increase during the lockdown period, probably due to growing practicality with the instrument and the modality of examination by both practitioners and patients. Therefore, a second phase (called KIT2) of the project has been planned. This phase will manage the shifting of digital remote patient contact systems from emergency to daily life.

In addition to the continuing use of the teleconsultation system during the KIT2 project, we aim to produce three different types of intervention: (i) a remodeling of the official Gemelli ART website, to be configured on the basis of a systematic collection of frequently asked FAQ questions; (ii) design of a chat-bot service to respond to patient needs; (iii) design of a specific mobile app for patient monitoring.

Future developments would have two phases: a first phase to catalyze the digital facilitation of patient communication through the remodeling of the website and the implementation of the chat-bot. In this way we would like to offer digital services that are synergistic with the communication that patients have with doctors, other health professionals, and administrative staff. The second phase will focus on the introduction of mobile applications for non-invasive remote monitoring of the patient’s clinical condition during treatment and the first follow-up period.

## 5. Conclusions

In our experience, the rapid implementation of a telemedicine system appears feasible even in pandemic emergency settings and well-accepted by patients. Moreover, this study opens up the opportunity for further applications of telemedicine in the field of radiation oncology.

## Figures and Tables

**Table 1:** Project phases.

Project Phases	
1	Creation of the multi-professional group
2	Planning visits with the new scheme: Value 1 = visit not deferrable and held in person Value 2 = visit postponed Value 3 = visit performed via email management and call Value 4 = visit performed via complete teleconsultation using teleconsultation system
3	Identification of the equipment necessary for the teleconsultation
4	Request for collaboration with the Patients’ Association for the buying of equipment
5	Start of televisit/teleconsultation
6	Telephone interviews for patient satisfaction surveys

**Table 2:** Questionnaire for telephone interview.

Questionnaire for Telephone Interview
Did you have any difficulties in managing the televisit or teleconsultation? [answers: yes/no] How much satisfied were you with the video-teleconsultation system? [answers in 5-likert scale]
When you realized that the pandemic could hamper your contact visit with your radiation oncologists, were you afraid your clinical needs would not reach properly your radiation oncologist before the televisit? [answers in 5-likert scale]
Do you think that, once the emergency is over, this type of service could be considered as an alternative of traditional on-site visits for selected patients? [answers in 5-likert scale]

## Data Availability

It is possible to request information on the data by contacting the first or corresponding author in the case of a reasonable request.
